# Understanding apathy: behavioral and neural correlates of apathy in neurodegenerative disorders

**DOI:** 10.1002/alz.095685

**Published:** 2025-01-09

**Authors:** Neke Nsor, Casey Brown

**Affiliations:** ^1^ Georgetown University, Washington, DC USA

## Abstract

**Background:**

Apathy, a common neuropsychiatric symptom of neurodegenerative disorders, involves a lack of motivation and reduced interest in positive activities, along with diminished concern for others. Deficits in basic aspects of emotional functioning including difficulties recognizing emotions (emotion recognition) and generating positive or negative emotions (positive and negative emotional reactivity) may underpin neuropsychiatric symptom clusters like apathy. Neural systems (i.e., anterior cingulate cortex, putamen, ventral striatum) that support emotional functioning may also be implicated in apathy. This study examined the behavioral and neural correlates of apathy in dementia.

**Method:**

The study included 434 care‐recipients with a variety of neurodegenerative disorders (n = 275), individuals at risk of transitioning to a neurodegenerative disease (n = 123), and healthy controls (n = 36). Care‐recipients underwent neuropsychological testing, neuroimaging, and clinical interviews to obtain a diagnosis. Caregivers reported on their care recipient’s emotional functioning using a well‐validated questionnaire and apathy was assessed using a clinical interview. We first tested whether the three aspects of emotional functioning (emotion recognition, positive emotional reactivity, and negative emotional reactivity) were associated with apathy using a linear regression. Then we examined the neural correlates of apathy with voxel‐based morphometry.

**Result:**

Greater apathy correlated with diminished positive emotional reactivity and emotion recognition, even after accounting for a number of covariates (i.e., care recipient age, gender, education, race, cognitive impairment, and diagnosis). Apathy was not significantly associated with negative emotional reactivity. At the neural level, increased apathy was associated with reduced grey matter in the ventral striatum and anterior cingulate cortex, while reduced emotion recognition was associated with reduced grey matter in the temporal cortex (Fusiform Gyrus & Temporal Pole). We did not find any significant association between grey matter volume and positive reactivity.

**Conclusion:**

Findings suggest that the abilities to recognize emotions and generate positive emotions are linked to apathy symptoms. Apathy in dementia may be associated with deficient activation in brain regions involved in reward and salience detection, whereas deficits in emotion recognition were linked to regions involved in face perception and social cognition. Further disentangling the associations between emotional functioning and neuropsychiatric symptoms may improve differential diagnosis and promote more targeted interventions.

